# Late HIV Diagnosis: Bad Medicine and Worse Public Health

**DOI:** 10.1371/journal.pmed.0040200

**Published:** 2007-06-12

**Authors:** Ronald O Valdiserri

## Abstract

The author discusses a new study that models the impact of the CDC's new recommendation of opt-out HIV testing in all health care encounters in the US for persons 13-64 years old.

It is estimated that there are some 252,000–312,000 Americans living with HIV yet unaware that they carry this deadly virus [1]. Once they develop symptoms of HIV disease, most will be diagnosed. But by then, their immune systems will have experienced permanent damage, and thousands will have inadvertently infected their partners. One analysis reckons that annual HIV transmission rates in the United States are some 3.5 times higher among people with undiagnosed HIV infection compared to those who are diagnosed [2], due to the fact that knowledge of positive HIV serostatus is associated with substantial decreases in high-risk sexual behaviors among those so diagnosed [3].

Although HIV remains a very serious medical concern, with the advent of improved antiretroviral treatments it is no longer the hopeless condition it was in the early 1980s. Yet late diagnosis of HIV infection remains a problem in the US three decades after the syndrome was first described, despite the substantial medical and public health benefits of early HIV diagnosis. In the US, about 40% of persons who were diagnosed with AIDS in 2005 had their first positive HIV test less than 12 months before their AIDS diagnosis [4]—meaning that they were infected with HIV for years prior to their initial diagnosis. Nor can late HIV diagnosis be simplistically ascribed to lack of access to health care. A variety of studies have shown that failure to diagnose HIV in a timely manner occurs even among persons who have regular access to health care [5–7].

## “Opt-Out” HIV Testing in Health-Care Settings

Arguably, addressing the persistent problem of late HIV diagnosis was the major driver in the recent push by the Centers for Disease Control and Prevention (CDC) for routine, opt-out testing in health-care settings, i.e., informing patients of the intent to perform HIV testing and inferring consent unless the patient declines [8]. Citing the time constraints of risk-assessment and counseling, CDC's 2006 guidelines also note that “prevention counseling should not be required with HIV diagnostic testing or as part of HIV screening in health care settings” [8].

**Linked Research Article**

This Perspective discusses the following new study published in *PLoS Medicine*:

Holtgrave DR (2007) Costs and consequences of the US Centers for Disease Control and Prevention's recommendations for opt-out HIV testing. PLoS Med 4(6): e194. doi:10.1371/journal.pmed.0040194

Scenario and cost-effectiveness analyses found that for the same programmatic cost, targeted counseling and testing would diagnose more people living with HIV and prevent more HIV infections than opt-out testing.

In a new study published in *PLoS Medicine*, David Holtgrave estimates the overall costs of CDC's routine HIV testing recommendations using standard methods of scenario and cost-effectiveness analysis [9]. His findings are provocative: in his scenarios, targeted HIV counseling and testing performed better than opt-out testing in terms of expense, number of newly identified patients, and prevention of HIV transmission [9].

Another salient feature of Holtgrave's analysis is his focus on the role of counseling in the context of HIV testing. He reminds readers that a randomized controlled trial sponsored by CDC in the late 1990s demonstrated that interactive client-centered counseling could reduce risk behaviors and the incidence of new sexually transmitted infections [10]. Although earlier CDC guidelines had already taken steps to streamline requirements for pretest counseling—acknowledging providers' need for flexibility based on client base, HIV prevalence in the health-care setting, and available resources [11]—the 2006 guidelines go even further. Prevention counseling is no longer identified as a requirement for HIV screening in health-care settings. One of Holtgrave's scenarios addresses this change, positing an increase in HIV incidence resulting from certain high-risk persons who, in the absence of counseling, might interpret negative test results as endorsing the “safety” of their behaviors.

## Implications of the Study

Perspectives on how best to encourage early diagnosis of HIV infection will continue to evolve as additional program implementation data and improved input parameters become available to further refine cost-effectiveness analyses. But it would be a serious mistake to narrow this multifaceted discussion to an artificial dichotomy of opt-out routine testing versus targeted HIV testing.

A broader, and perhaps more relevant, question worthy of further study is “How can we configure and sustain health-care systems that are capable of promoting and incentivizing necessary prevention services such as early HIV diagnosis?” [12]. These systems should be able to address the needs of individual patients, such as those patients who might require more time to accept HIV testing, even in an opt-out scenario, or those patients who receive a negative HIV test result, but require active intervention to facilitate changes in ongoing high-risk behaviors. In our current systems of care, limited provider time and competing demands are well-recognized obstacles to the optimal delivery of a host of clinical preventive services—not just HIV counseling and testing [13]. And although prioritization of clinical preventive services at a population level is a practical strategy to allocate scarce prevention resources [13], it may not serve the prevention needs of individuals or subgroups.

Future research on the timely diagnosis of HIV infection must include operational studies and systems analyses that explicitly evaluate the impact of changes in the design of health-care delivery systems (including referral systems) on the receipt of lifesaving services like HIV testing and, when needed, ongoing risk reduction counseling [14].

## Abbreviations

CDC: Centers for Disease Control and Prevention
